# Bio-multifunctional noncovalent porphyrin functionalized carbon-based nanocomposite

**DOI:** 10.1038/s41598-021-86119-z

**Published:** 2021-03-23

**Authors:** Navid Rabiee, Mojtaba Bagherzadeh, Amir Mohammad Ghadiri, Yousef Fatahi, Nafiseh Baheiraei, Moein Safarkhani, Abdullah Aldhaher, Rassoul Dinarvand

**Affiliations:** 1 Department of Chemistry, Sharif University of Technology, Tehran, Iran; 2 Department of Pharmaceutical Nanotechnology, Faculty of Pharmacy, Tehran University of Medical Sciences, 14155-6451 Tehran, Iran; 3 Nanotechnology Research Center, Faculty of Pharmacy, Tehran University of Medical Sciences, 14155-6451 Tehran, Iran; 4 Universal Scientific Education and Research Network (USERN), 15875-4413 Tehran, Iran; 5 Department of Anatomical Science, Faculty of Medical Sciences, Tarbiat Modares University, Tehran, Iran

**Keywords:** Biotechnology, Chemistry

## Abstract

Herein, in a one-pot method, the reduced graphene oxide layers with the assistance of multiwalled carbon nanotubes were decorated to provide a suitable space for the in situ growth of CoNi_2_S_4_, and the porphyrins were incorporated into the layers as well to increase the sensitivity of the prepared nanostructure. The prepared nanocomposite can establish π–π interactions between the genetic material and on the surface of porphyrin rings. Also, hydrogen bonds between genetic domains and the porphyrin’ nitrogen and the surface hydroxyl groups are probable. Furthermore, the potential donor–acceptor relationship between the d^7^ transition metal, cobalt, and the genetic material provides a suitable way to increase the interaction and gene loading , and transfections. The reason for this phenomenon was optimized to increase the EGFP by up to 17.9%. Furthermore, the sensing ability of the nanocomposite towards H_2_O_2_ was investigated. In this regard, the limit of detection of the H_2_O_2_ obtained 10 µM. Also, the in situ biosensing ability in the HEK-293 and PC12 cell lines was evaluated by the addition of PMA. The nanocomposite showed the ability to detect the released H_2_O_2_ after adding the minimum amount of 120 ng/mL of the PMA.

## Introduction

Carbon-based nanomaterials have been considered recently for different biomedical applications due to their unique physical and chemical properties. Recently, The use of carbon-based nanomaterials due to their outstanding biocompatibility as well as biodegradability is increasing. Also, making multi-functional synthetic materials can be a smart strategy to advance studies and technologies. In this regard, cost-effectiveness should be considered, along with high accuracy and sensitivity. Meanwhile, studies based on different nano-bio sensors have expanded significantly due to the importance of analytical techniques in various industries and human health. Nevertheless, complexity of synthetic methods in some studies make this type of research away from the competitive environment of different industries^1, 2^.

The importance of the presence of hydrogen peroxide (H_2_O_2_) is not entirely unknown to scientists. However, it has been considered a “necessary evil” for different kinds of mammalian cell signaling and other reactions. The H_2_O_2_ is the most routine and known represents reactive oxygen species (ROS). The presence of H_2_O_2_ is responsible for a different range of cellular phenomenona including cellular proliferation, migrations, and differentiation. Still, the overgeneration of that would indeed lead to disruption of the redox homostasis of cells and, in the following unwanted bio tissues oxidative stress. Finally, it can lead to different kinds of cancer, aging, cardiovascular dysfunctions, and Alzheimer’s and Parkinson’s diseases. Therefore, in situ measuring and monitoring the real-time concentration of H_2_O_2_ by a simple, low-cost, and ultra-sensitive nanosystem from living organisms is very important^3, 4^.

In this regard, a wide range of electrochemical and optical biosensors have been designed, developed, that studied for early and post-detection of diseases and biological phenomenon. These systems are based on the detection of cellular secreted H_2_O_2_ in assistance of using metal oxide nanoparticles (Au, Mo, Ag, Zn), carbon nanotubes (CNTs), reduced graphene oxides (rGOs), and protein-based microarrays. All of those mentioned raw materials have their advantages, including simplicity and acceptable sensitivity. However, disadvantages including lack of accuracy, time-consuming analysis, low-sensitivity, and hard tenability necessitae working on different types of materials and nanocomposites^5, 6^.

Another emerging application of carbon-based nanomaterials is the gene delivery systems. Two of the essential features that this material should have for gene delivery applications are a small size and the ability to interact with genetic material and form a stable complex^7–11^. Zeta-positive polymers are commonly used for this purpose, and there is no comprehensive study on the use of nanocomposites in this field. However, this is a positive charge that can lead to a relatively strong interaction, but physical cracks on the material surface can lead to good interaction. In this research, we try to use the knowledge of chemistry. With the help of combining various structures, we must prepare a nanocomposite with many steps with different sizes on the surface that can lead to a stable interaction with genetic material.

In this study, we aimed to develop a promising and fully optimizable 3D carbon-based nanocomposite being decorated with magnetic and other types of nanoparticles by the help of porphyrins. These carbon-based nanomaterials have acceptable biocompatibility and minimum cytotoxicity in a varied range of cell lines. In this work, we aim to molecular engineer the carbon-based layer (rGO) to provide a suitable space for in situ and one-pot growth of CoNi_2_S_4_ by introducing different mechanistic pathways. In this regard, the multiwalled carbon nanotube (MWCNT), which can improve the cellular penetration with low immunogenicity, is incorporated between the rGO layers to increase the space between them. Besides, investigating the possible donor–acceptor electronic interaction between the d^7^ transition metal (Cobalt) and the genetic material (which has electron-rich and electron-poor sites) could increase the interactions enhancing the gene delivery efficiency.

**Figure 1:**
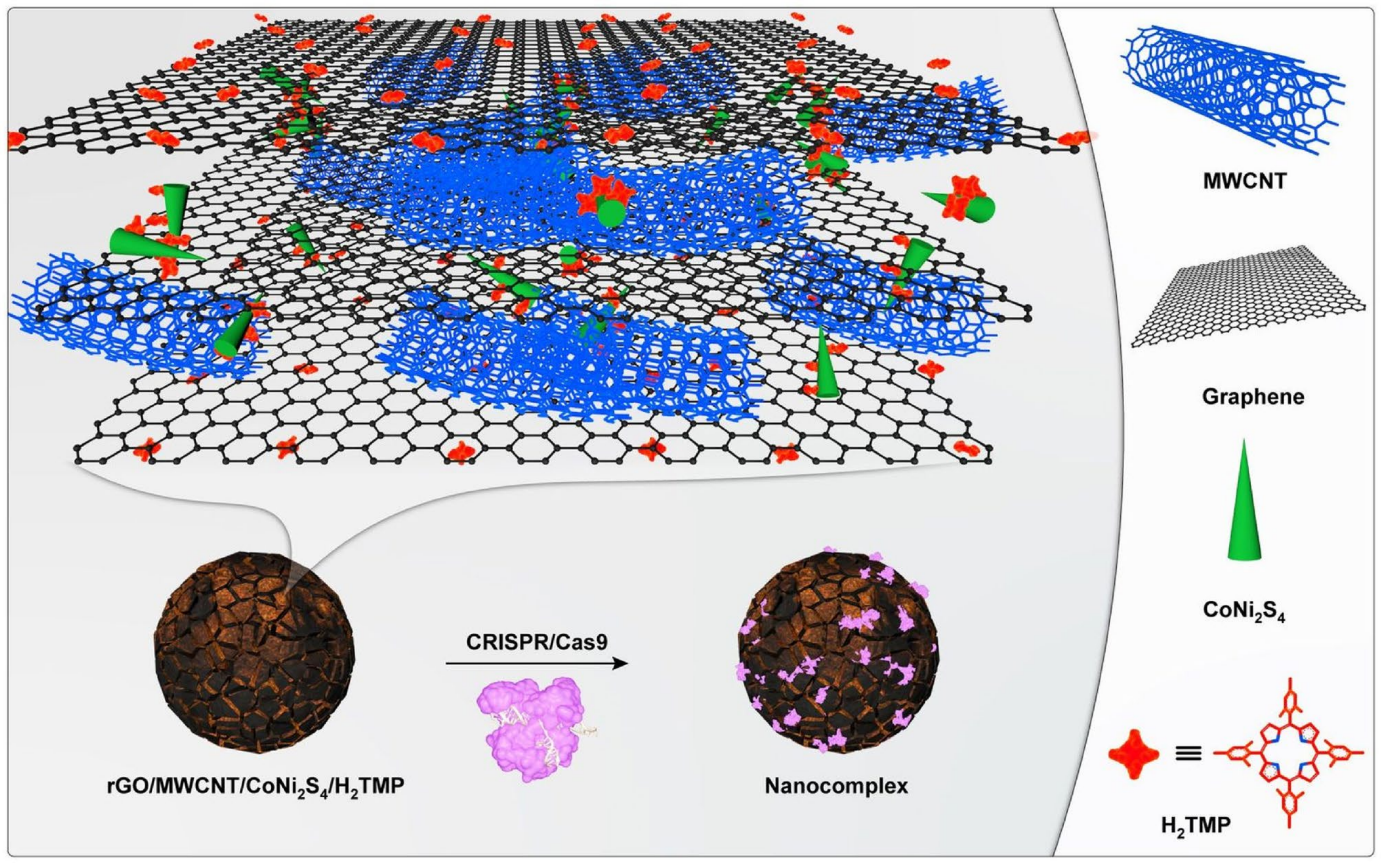
The schematic illustration of the synthesized nanosystem.

Along with that, CoNi_2_S_4_ has a semi-cylindric shape that can adsorb the domain of any genetic materials with the help of space-selective interactions. For the first time, we developed the fully biocompatible nanocomposite from non-biocompatible compartments, which have a large surface area and conductivity. This work also represents that the synthesized nanocomposite directly can improve the cellular uptake and is used as a suitable and highly-promising candidate as a sensing nano-platform for conformal cell monitoring the particular mechanical deformations. The schematic illustration of the nanosystem is observed in Fig. 1. In this work, we aimed to design and synthesize of bio-multifunctional nanocomposite with the ability to make different physical interactions with the genetic material. This is the first study to provide and represent a new type of interaction, and it should be noted that more research is needed to prove the other capabilities of this nanocomposite. In addition, there are lots of physical and chemical approaches in order to functionalized graphene and graphene-based nanomaterials for drug/gene delivery systems (Table 1); however, till now, there are no reports regarding the use of semi-cylindric shape nanoparticles along with the other π–π and hydrogen bonding interactions at the same time.

**Table 1:** A literature survey summary regarding the physical and chemical approaches in order to modify the graphene-based nanomaterials for drug/gene delivery applications.

Functionalization method	The precursors and/or the mechanisms	References
Covalent	Fe_3_O_4_	^12^
	Chitosan	^13^
	Sulfonic acid	^14^
	Dextran	^15^
	Gelatin	^16^
	Folic acid	^17, 18^
	Polyethylene glycol	^19^
	Poly-L-lysine	^20^
	Polyethyleneimine	^21^
	Amphiphilic copolymers	^22^
	Polyacrylic acid	^23^
	Poly(vinyl alcohol)	^21^
	Poly(N-isopropylacrylamide)	^24^
	Polysebacic anhydride	^25^
Non-covalent	π–π interactions	^26, 27^
	Electrostatic	^28, 29^
	Coordination bondings	^30^
	Van der Waals	^31, 32^
	Hydrogen bonding	^33^
	π–π interactions + electrostatic + van der Waals interactions + hydrogen bonding	This work

## Experimental section

### Materials

The potassium permanganate (KMnO_4_), graphite powder, sulfuric acid (H_2_SO_4_, 98%), hydrogen peroxide (H_2_O_2_, 30% v/v), hydrochloric acid (HCl, 36%), Mesitylene (98%), Mesitaldehyde, Chloroform, trifluoride-diethyl ether, pyrrole, *p*-chloranil, Ni(NO_3_)_2_.6H_2_O, Co(NO_3_)_2_.6H_2_O, thiourea, dimethylformamide (DMF), multiwalled carbon nanotubes (MWCNTs, purity > 90%, length < 9 µm , OD = 10–20 nm) and sodium acetate anhydrous (NaAc) were purchased from Sigma-Aldrich, Germany.

### Synthesis of H_2_TMP

The synthesis of porphyrin was conducted based on the literature^34^. For the synthesis of H_2_TMP, briefly, 10 mmol mesitaldehyde and 10 mmol distilled pyrrole were added to 100 mL chloroform. The solution was stirred for 10 min as well as purging the N_2_. Then, 3.3 mmol of boron trifluoride-diethyl ether (10^–3^ M) was added to the reaction. The solution was stirred at room temperature for 1 h and oxidized with an excess amount of DDQ, followed by absorption spectrophotometry. Then, 7.5 mmol of *p*-chloranil was added to the reaction mixture and refluxed for 1 h. Finally, the reaction mixture was cooled to room temperature, and 3.3 mmol of trimethylamine was added following by rotary evaporation of the solution. The product was washed several times with ultrapure water and methanol.

### One-pot synthesis of rGO/MWCNT/CoNi_2_S_4_/H_2_TMP

The desired nanocomposite was prepared by an in situ one-pot solvothermal procedure^35^. The one-pot synthesis method was introduced for the growth of CoNi_2_S_4_ in the physical space between the rGO sheets, which were provided by the MWCNT’s. Furthermore, the reduced graphene oxide (rGO) was synthesized by the modified hummer’s method^36^. Briefly, 50 mg of the MWCNT and 100 mg of the rGO were dispersed in ethylene glycol (50 mL) and sonicated for about 5 min. In the next step, 55 mL of IPA/water (1:1) was added, and the final mixture was sonicated (4.5 h; 40 kHz frequency). The obtained mixture was centrifuged (15 min; 2000 rpm), and the supernatants were removed and filtered (the non-reacted exfoliated materials) by an additional centrifuge (15 min; 3000 rpm). Furthermore, Ni(NO_3_)_2_.6H_2_O (2 mmol), Co(NO_3_)_2_.6H_2_O (1 mmol), and thiourea (9 mmol) were added to the above solution (40 mL) and stirred for about 20 min. After that, ammonia (1 mL) was added dropwise to the solution and sonicated (60 min; 40 kHz frequency). In another experiment, the synthesized H_2_TMP (5 mg) was dispersed into DMF (12 mL) and sonicated in a dark place for 20 min, and then added to the first solution. The obtained mixture was transferred to a 200 mL autoclave (Teflon-lined stainless steel) and kept at 175 °C for a day (24 h). The obtained black powders were washed a few times with ultrapure water and ethanol and freeze-dried for 36 h.

### Cell evaluations

#### MTT and LDH assay

Before cell culture, samples were sterilized using ultraviolet exposure followed by washing with ethanol (75%) and PBS solution. Cytocompatibility assessment was performed using MTT (3-[4,5-dimethylthiazol-2-yl]-2,5-diphenyltetrazolium bromide) (MTT, Sigma) colorimetric assay at 24, and 48 h. PC12 cells (ATCC CRL-1721), HEK-293(ATCC CRL-1721), HeLa (ATCC CCL-2), and HepG2 (ATCC HB 8065) were used for this experiment. Briefly, 1 × 10^5^ cells/well were cultured on the synthesized nanocomposite substrate in Dulbecco’s Modified Eagle’s Medium (DMEM, Gibco) containing 100 IU/ml penicillin, 100 IU/ml streptomycin (Invitrogen), and 10% fetal bovine serum (FBS; Gibco) and incubated at 37 °C at 5% CO_2_. At each time point, 100-μL MTT solution (5 mg/mL in PBS) was added to each well. After 4 h. Incubation, the medium was removed, and formazone precipitates were dissolved in dimethyl sulfoxide (DMSO; Sigma-Aldrich). The optical absorbance was measured at 570 nm using a microplate Elisa reader (ELX808, BioTek). At least three samples were averaged to calculate each time point.

In onrder to conduct the lactate dehydrogenase (LDH) leakage assay, HepG2 cells were cultured with the density of 1 × 10^5^ cells/well for 24 h. The grown cells were treated with the nanocomposite by the procedure same as the MTT assay. After the incubation, the media of cell culture was transferred to a microtube, and centrifuged at 12,000 rpm for 10 min. In the next step, 100 μL of of the working reagent (contains sedum pyruvate (2.5 mM) and NADH (0.2 mM)) was prepared, and 10 μL of the supernatant was added to it. The optical absorbance was measured at 340 nm using a microplate Elisa reader (ELX808, BioTek). At least three samples were averaged to calculate each time point.

#### Biosensor assay

For this assay, both PC12 and HEK-293 cell lines were cultured in conditions mentioned above. First, 1 × 10^5^ cells/well were seeded on the synthesized nanocomposite. After about 24 h, the implanted cells were evaluated for the H_2_O_2_ releasing the study, based on the mechanism of destruction of porphyrin (TMP) with the released H_2_O_2_.

### Loading CRISPR and tagged DNA on the surface of modified Materials

The different procedures of the loading of CRISPR and tagged DNA on the surface of modified materials were reported previously^37, 38^. Initially, the modified nanocomposite was dispersed with a concentration of 22 mg mL^−1^ in the ultrapure water. Then, different weight ratios of the modified nanocomposite and CRISPR plasmid (pCRISPR) formulated through nanocomposite with different concentrations to an exact volume of the solution of CRISPR, and the homogenous solutions were prepared. Briefly, the weight ratios of 1, 10, 20, 30, 50, and 100 of the nanocomposite to pCRISPR were prepared and incubated for about 30 min to form the ultimate nano complexes. The entirely physical interactions between the active sites of the nanocomposite and the pCRISPr and,or DNA leads to make an acceptable and suitable final nano complex.

### Size and surface charge analysis

The size of the nanocomposite (NC)/CRISPR/Cas9 (CC) nanocomplex was investigated using dynamic light scattering on a Zetasizer Nano ZS (Malvern Instruments, UK). The investigations were performed at standard angles of 25° and 173°, and each measurement was verified three times. In this technique, the size of the nanocomplex is reported as the mean diameter achieved by the refractive index and viscosity of water correlation functions cumulants analysis. For aggregation kinetics measurements, the final nanocomplexes were diluted with a ratio of 1:4 with double HBSS at the neutral pH, which obtained isotonic formulations that are identified as the technique used in the transfection experiments. For zeta potential investigation, doppler velocimetry was applied on the same instrument.

### Statistical analysis

All of the statistical analysis was performed by one-way analysis of variance (ANOVA) followed by OriginPro 9.1 software compatible tests using Bonferroni post-hoc tests. Also,, all data represented are the mean ± SD of at least n = 3 independent sets of experiments.

## Results and discussion

### Synthesis of the nanocomposite

**Figure 2:**
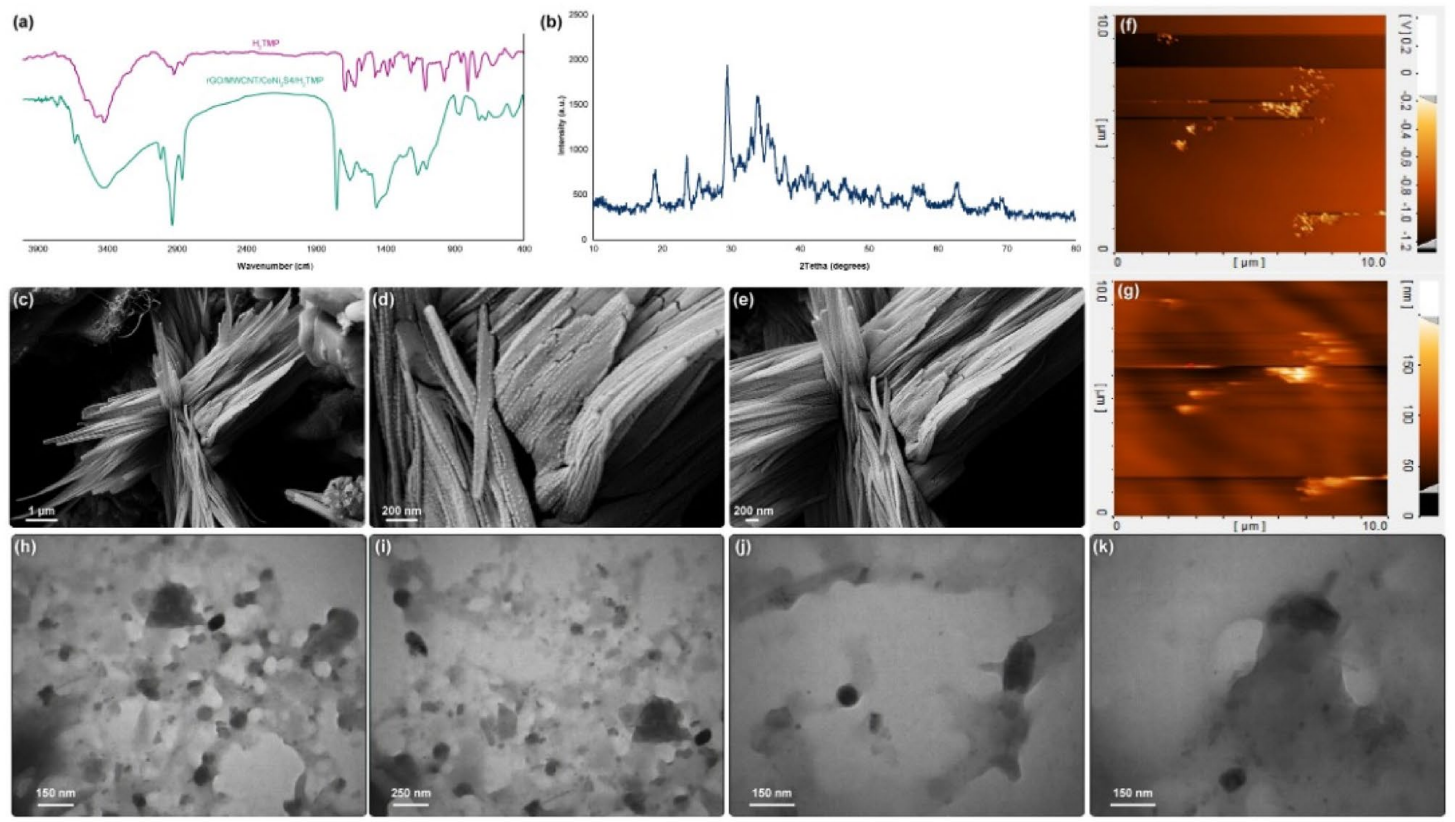
FTIR (**a**), XRD (**b**), FESEM (**c**–**e**), AFM (**f**,**g**) ,and TEM (**h**–**k**) of the synthesized nanocomposite.

Based on the FTIR results (Fig. 2a), a broad peak at around 3400 cm^−1^ represents stretching vibrations of N–H and the complex peaks in the range of 900–1200 cm^−1^ assigned to the bending vibrations of N–H. Furthermore, the weak peaks in the range of 2850–3050 cm^−1^ correspond to the stretching vibrations of C–H of the porphyrin rings. The 1400 cm^−1^ peak assigns to the C=N pyrrole ring vibrations. Also, the complex peaks in the range of 600–800 cm^−1^ represent the main vibrational peaks of the porphyrins. All of these observations represent the successful synthesis of H_2_TMP^39^. For the synthesized nanocomposite, all of the sharp and indicates absorption peaks of the H_2_TMP observed clearly, which presents the proper incorporation of the porphyrin to the nanocomposite structure. Besides, the fingerprint absorption peaks of CoNi_2_S_4_, which are 3451 cm^−1^, 1630 cm^−1^, 1370 cm^−1^, 901 cm^−1^, and 610 cm^−1^ corresponds to the stretching and bending vibrations of the water compartment (O–H) on the surface of the Co (Co–O–H), C–O–C vibrations, N–C=S and C=S vibrations of the thiourea, respectively^40, 41^. These peaks indicate the successful in situ and one-pot synthesis of nanocomposite along with CoNi_2_S_4_. It should be noted that the synthesis method of Ni_3_S_2_ is the same as the CoNi_2_S_4_ with some minor modifications. However, the fingerprints of the Ni_3_S_2_ are 2217 cm^−1^, 2108 cm^−1^, 1870 cm^−1^, and 618 cm^−1^, which have differences with the present spectrum^42–44^. The synthesized nanocomposite has minimal covalent interactions with the H_2_TMP; if the cobalt had any covalent interactions with the porphyrin, some major and sharp peaks at 1099 cm^−1^, 920 cm^−1^, and also 802 cm^−1^ should be observed^45–47^. In the FTIR spectrum of the synthesized nanocomposite compared to the H_2_TMP, those absorption peaks are not observed critically; therefore, there are no significant and strong covalent interactions between them. So, the porphyrin ring is free of any coordinated metal, or even partially free, which leads to the hypothesis that the metal is located at the bottom of the porphyrin plane and only interacts with the central ring of porphyrin due to the high density of electrons. Therefore, the center and top of the porphyrin plane are free of any coordination, which is highly suggested for any physical interactions with the biomolecules for gene delivery applications.It ia also suitable for chemical and biological reagents for biosensor use. The XRD pattern (Fig. 2b) represents the indicative peaks at 2θ = 77.6°, 68.8°, 64.6°, 55.0°, 50.2°, 47.1°, 38.0°, 31.4°, 26.6° and 16.2°, which is the proof of the successful in situ synthesis and incorporation of CoNi_2_S_4_ in the nanocomposite^48, 49^. Those peaks are in good agreement with the JCPDS 00-024-0334^50^. The broadening in the diffraction peaks corresponds to the presence of H_2_TMP as well as the byproducts^51, 52^. In this study, the aim was to provide a facile, one-pot, and tunable synthesis method with potential biomedical applications. Therefore, the byproducts do not consider as a contradiction for this type of study. Besides, FESEM (Fig. 2c–e) and AFM (Fig. 2f,g) analysis were conducted. It has been depicted that; the nanocomposite has the 2D nanosheet subunits as well as 1D tubular framework nanostructure, which is because of the presence of CoNi_2_S_4_ in the and on the nanostructure of carbon-based nanomaterial^53, 54^. In addition, another strong fact regarding the presence of CoNi_2_S_4_ was provided by elemental mapping (Figure S1). Based on this analysis, the presence of CoNi_2_S_4_ compared to the carbon-based substrate, has the higher intensity; and among the different elements on the CoNi_2_S_4_, sulfur has the highest intensity, which is another fact regarding the successful synthesss of this structure. Also, the presence of H_2_TMP leads to more aggregations and crossling of different parts of the nanocomposite^55–57^. These crosslinking leads to the formation of different open and void space channels, which could interact with any types of materials, including genetic materials and CRISPR/Cas9. These voids and free channels may enable different active sites in the porous and hierarchical nanostructure that can act as electron/ion transport routes and increase the kinetic of any types of reactions and interactions. These open and void space channels can be observed in the TEM (Fig. 2h–k) images; also, the structure of the irregular sheets of the rGO is observed. Based on the both FESEM and TEM images, the size of the CoNi_2_S_4_ is withing the range of 100–200 nm in the length, and 5–20 nm in diameters. These sizes are normal and based on the literature, the size of the synthesized CoNi_2_S_4_ are varied in the range of ± 40% of the mentioned numbers. However, in this study, due to the presence of carbon-based substrate, and the porphyrins, substantial aggregations would leads to considerable physical interactions between the compartments. Therefore, analyzing the exact size of each compartment is not applicable in this study. The aim of this study was not to provide a clear, sharp and high-quality material, but the aim was to design and provide a low-cost, one-pot and environmentally-friendly synthesis method with the applicable biomedical products.

### H_2_O_2_ biosensor performance

**Figure 3:**
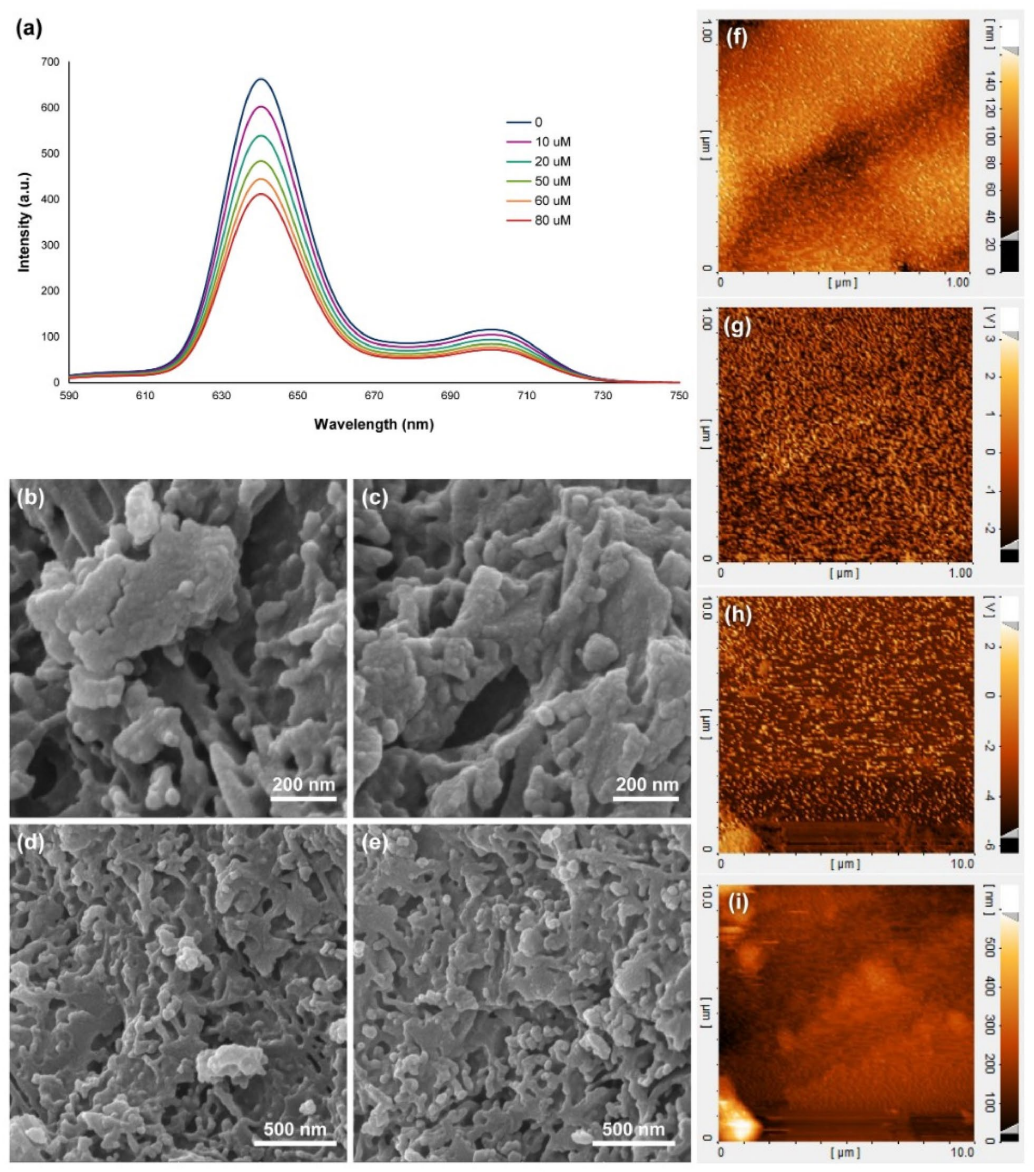
Fluorescence emission spectra of the synthesized 3D nanocomposite in the presence of different concentrations of hydrogen peroxide (**a**), FESEM (**b**–**e**), and AFM (**f**–**i**) images of the synthesized 3D nanocomposite after exposure to the hydrogen peroxide.

The hydrogen peroxide detection performance from a living cell with an on-line and in situ experiment was conducted using the synthesized nanocomposite (Fig. 3a). Based on the results, there is a decline in the fluorescence emission of the nanocomposite in the presence of different concentrations of H_2_O_2_ up to 80 µM, which led to hydroxylation of the meso- position of the porphyrin rings, then, the oxidative ring reaction starts from this position^58–60^. The estimated linear detection range was calculated to be from 10 µM to 25 mM (Figure S2), with a considerable limit of detection (LOD) of about 10 µM (S/N = 3). These results could be because of the space-hindrance porphyrin structure, H_2_TMP, with the large substituents that inhibit the potential chelation or coordination. There is no active metal in our system, and also it cannot interfere with any type of material in this system. Therefore, great LOD and linearity could be expected. By comparing this study’s results with the same scope works on even electrochemical biosensors, our designed biosensor has more comprehensive linear range with a significant LOD and considerable accuracy. Based on the literature, the interaction results in changes in the structural geometry of porphyrin from the flat state. This curved structure disrupts the electron structure of porphyrin, which reduces the porphyrin stability and increases the oxidation rate of the meso-position^61–64^. Also, based on the mentioned mechanism, electron-donating groups on the meso-position of the porphyrin ring increase the electron density on the porphyrin ring, followed by the increase in the oxidation rate of the meso-position and degradation of the porphyrin, and the electron-withdrawing groups decrease the electron density on the porphyrin ring, as well as a decrease in the oxidation rate of the meso-position and degradation of the porphyrin. Consequently, meso-position oxidative rate of the H_2_TCPP is lower than the meso-position oxidative rate of the H_2_TPP, and the highest meso-position oxidative rate belongs to the H_2_TMP. Our designed nanocomposite is tunable to be optimized based on different analytes and different cellular conditions. These optimizations can be conducted by changing the substitutions on the porphyrin ring from electron withdrawing to electron donating groups and conversely. After completing the experiment regarding the biosensor performance, FESEM (Fig. 3b–e) and AFM (Fig. 3f–i) analysis were conducted on the synthesized 3D nanocomposite. These results showed the structural deformation on the surface of that because of hydrogen peroxide and destroying the porphyrins. Based on the results, this is the first study in the study and monitoring of the hydrogen peroxide release from the living cells in the quenching of the porphyrin's fluorescence spectra. However, some efforts were made based on highly-stretchable electrochemical biosensors with the best limit of detection of 12 µM and a linear range of 40 µM to 15 mM by using gold nanowires^65^. The fluorescence one had the limit of detection of 200 µM by using organic hydrogels^66^.

### In situ and real-time monitoring of hydrogen peroxide from the living cell

**Figure 4:**
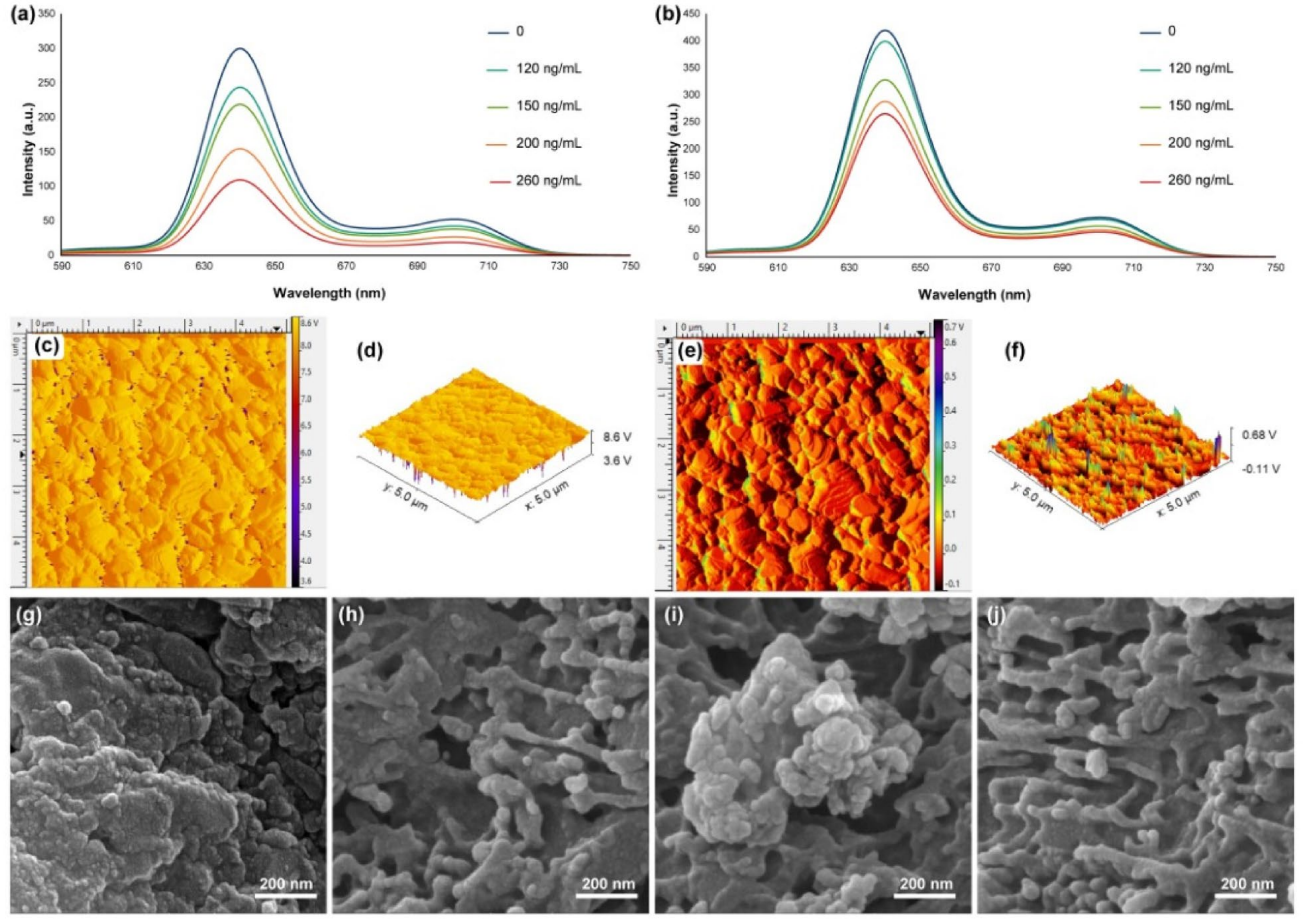
Fluorescence emission spectra of the synthesized 3D nanocomposite in the presence of different concentrations of PMA added to the HEK-293 (**a**) and PC12 (**b**) cell lines. 2D (**c**) and 3D (**d**) AFM images and (**g** and **h**) FESEM images of the synthesized 3D nanocomposite after removing from the PC12 culture and exposure to the hydrogen peroxide; 2D (**e**) and 3D (**f**) AFM images and (**i** and **j**) FESEM images of the synthesized 3D nanocomposite after removing from the HEK-293 culture and exposure to the hydrogen peroxide.

HEK-293 and PC12 cells were selected to assess the potential of using the synthesized nanocomposite in the living cells to determine and detect the released hydrogen peroxide. Figure 4 shows the fluorescence spectroscopy results of the nanocomposites that have been exposed to the cells and their hydrogen peroxides released. If the cells do not have significant and suitable adhesions, therefore, the fluorescence spectra do not show trends of the decline of emission in the presence of a different concentration of added PMA. These results support the significant adhesion and growth of the mentioned cells (mammalian cells) on the synthesized 3D nanocomposite. However, the nanocomposite’s cytotoxicity towards the PC12 cells does not change the ratio of adhesion and the growth of them on the substrate. It should be noted that, based on the literature^67–69^, the similar nanocomposites, mainly cobalt-based^70–72^, cannot be well used to identify and detect different parameters for in situ analysis because of their considerable cytotoxicity as well as their low cellular adhesion. Still, in this study, based on fluorescence images, it can be concluded that the synthesized nanocomposite has acceptable cell adhesion that can perform this process.

To monitor the hydrogen peroxide release from living cells, the phorbol 12-myristate 13-acetate (PMA) was used as a standard and routine stimulus agent to express hydrogen peroxide^73–75^. By adding this stimulus agent, PMA, a series of signaling pathways activated, and the hydrogen peroxide released from the living cells in a non-controlled manner. The hydrogen peroxide release mechanism is based on the oxidation of oxygen by NADPH oxidase and generating the O_2_^-^. To optimize the release of hydrogen peroxide upon the addition of PMA, a series of studies were designed and optimized based on the presence of HEK-293 and PC12 cell lines. Based on the results on the HEK-293 cell line (Fig. 4a), upon addition of 120 ng mL^−1^ of PMA to the cultured cell lines on the 3D nanocomposite, a distinctive decline in the fluorescence spectra of the media was observed, which was an equivalent phenomenon same as the addition of 20 µM of hydrogen peroxide. The maximum fluorescence intensity of the 3D nanocomposite decreased up to below a third compared to the absence of PMA. Furthermore, the PC12 cell line (Fig. 4b) represents not too much decrease in the presence of PMA, which could be considered a promising result based on the designing of a semi-selective fluorescence biosensor towards the HEK-293 cell line. Also, the maximum decrease of the fluorescence intensity in the presence of up to 260 ng mL^-1^ of PMA is equivalent to 40 µM of hydrogen peroxide. Therefore, this biosensor is not sensitive to both of the PC12 and HEK-293 cell lines. However, this is sensitive and accurate for analyzing released hydrogen peroxide from the HEK-293 cell line. Furthermore, the morphology of the synthesized 3D nanocomposite was investigated after removing from the PC12 and HEK-293 cultures via AFM (Fig. 4c–f) and FESEM (Fig. 4g–j), which have been shown that the surface morphology does not change significantly. However, the bulk breaks down into smaller pieces because of the in situ hydrogen peroxide and the nanocomposite’s cellular environment. Interestingly, by comparing the results of Fig. 4c–f with Fig. 3f–i, it is understood that in the presence of pure hydrogen peroxide, the surface has changed. Still, in the presence of hydrogen peroxide produced by PC12 and HEK-293 cells, the surface has not changed much, and only bulk has changed significantly. These results could indicate the specific intracellular and in situ measurement ability of this nanocomposite, which can even be used more than once. Based on the literature, this is the first work on the application of nanocomposites, especially with the assistance of the porphyrins, in real-time monitoring of the generated hydrogen peroxides from living cells. Most of the works in the literature are focused on the electrochemical devices (with the limit of detection of 20–100 µM)^76, 77^, chemiluminescence nanoprobes (with the detection limit of 60–300 µM)^78^, and some unique chemical mechanisms (with the detection limit of above of 300 µM)^79, 80^; therefore, the results of this study with the limit of detection of lower as 20 µM could be considered promising.

### Cell evaluations

**Figure 5:**
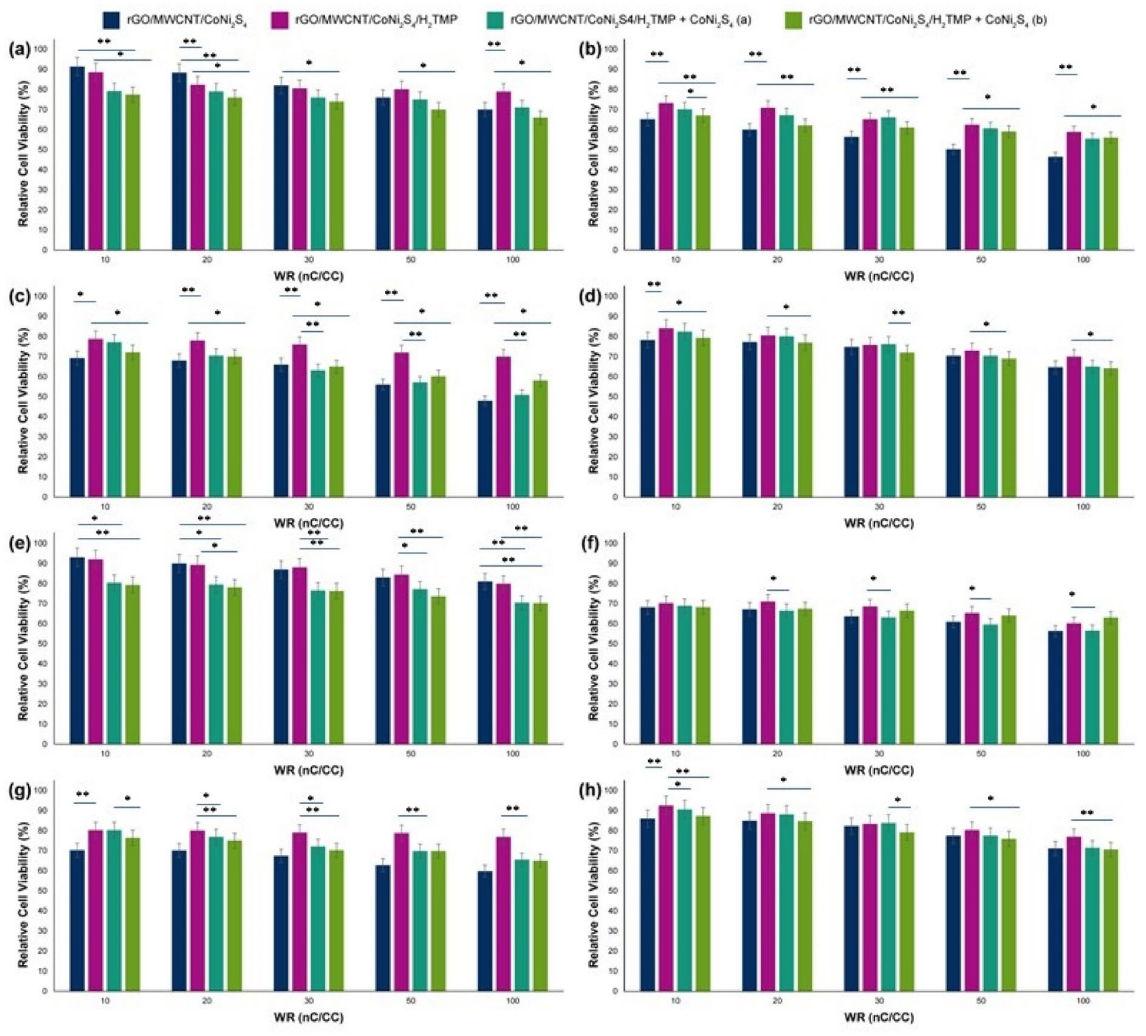
MTT results of the rGO/MWCNT/CoNi_2_S_4_, rGO/MWCNT/CoNi_2_S_4_/H_2_TMP, rGO/MWCNT/CoNi_2_S_4_/H_2_TMP + CoNi_2_S_4_ (**a**) and rGO/MWCNT/CoNi_2_S_4_/H_2_TMP + CoNi_2_S_4_ (**b**) on the (**a**) HEK-293, (**b**) HeLa, (**c**) HepG2 and (**d**) PC12 cell lines after treatment time of 48 h; and rGO/MWCNT/CoNi_2_S_4_, rGO/MWCNT/CoNi_2_S_4_/H_2_TMP, rGO/MWCNT/CoNi_2_S_4_/H_2_TMP + CoNi_2_S_4_ (**a**) and rGO/MWCNT/CoNi_2_S_4_/H_2_TMP + CoNi_2_S_4_ (**b**) on the (**e**) HEK-293, (**f**) HeLa, (**g**) HepG2 and (**h**) PC12 cell lines after treatment time of 24 h; (**a**) represents for 10 mg mL^−1^ and (**b**) 20 mg mL^−1^ concentrations of the excess CoNi_2_S_4_. *p value < 0.05 and **p value < 0.01.

Before any transfection studies, cell viability against different cell lines was investigated following 24 and 48 h. treatment with the nanocomposite (Fig. 5). As can be seen, carbon-based nanomaterial and the CoNi_2_S_4_ part have the HEK-293 cell viability of about 91% ± 1.30 in low WR of nC to CC (WR of 10) and 70% ± 1.09 in the highest WR of nC to CC (WR of 100). By the addition of porphyrin (H_2_TMP) to the nanocomposite, the cell viability decreased from 91% ± 1.30 to 88% ± 1.12 for the WR of 10 and increased from 70% ± 1.09 to above of 78% ± 1.10 for the WR of 100. The corresponding value, then, decreased significantly (p ≤ 0.001) to 75% ± 1.10 and 67% ± 1.05 for the WR of 10 and 100, respectively. After the optimizations, the only parameter that changes during the process is the zeta potential and hydrodynamic size of the particles; however, the zeta potential range of changes is minimal. Therefore, the only parameter that can correspond to the relative cell viability changes is the hydrodynamic size. Interestingly, the increase of the cell viability by addition of H_2_TMP for the high WR of nC to CC (50 and 100), would be because of decreasing the particle size in these WR’s substantially. The same trend was observed for HeLa, HepG2, and PC12 cell lines, as well. Briefly, the cell viability of rGO/MWCNT/CoNi_2_S_4_ after addition of H_2_TMP increased from 65% ± 1.03 to 73% ± 1.08 for the WR of 10 and from 46% ± 0.93 to 58% ± 1.01 for the WR of 100 after treatment with the HeLa cell line. Both values experienced a significant drop to 69% ± 1.05 (p ≤ 0.001) and 58% ± 1.01 (p ≤ 0.005) after the addition of CoNi_2_S_4_, respectively. Also, cell viability increased from 69% ± 1.05 to 78% ± 1.10 for the WR of 10 and from 48% ± 0.93 to 70% ± 1.05 for the WR of 100 against the HepG2 cell line. After treatment with the PC12 cell line, cell viability increased from 78% ± 1.09 to 84% ± 1.20 for the WR of 10 and from 64% ± 1.04 to 70% ± 1.05 for the WR of 100. In fact, adding more CoNi_2_S_4_ in the forms of nanoparticles to the nanocomposite, the cell viability decreased in all of the cell lines and all of the WR’s; however, this decrease was more obvious for the HEK-293 and HepG2 cell lines. It should be noted that the same trend was observed for 24 h of treatment time as well. Based on the literature, there is no report regarding the cell viability of the synthesized nanocomposite and even similar components; however, the cell viability of the nanocomposites and their components in comparison to the similar inorganic compounds based on Cobalt (cell viability on the HEK-293 and PC12 is around 50–70%)^81, 82^, Nickel (cell viability on the HEK-293 and PC12 is around 49–67%)^83^, and porphyrins (cell viability on the HEK-293 and PC12 is around 54–79%)^84, 85^, has higher percentages. Also, the LDH results (Figure S3) confirmed the MTT assay results, and showed that the membrane of the cells were approximately intacts, however, the interactions were remained strongly. In addition, it should be noted that, further assay regarding the exact cellular and molecular mechanisms of these MTT and LDH in the presence of these types of nanocomposites, and derived nanomaterials should be conducted before any in vivo trials.

### CRISPR/Cas9 investigations

**Figure 6:**
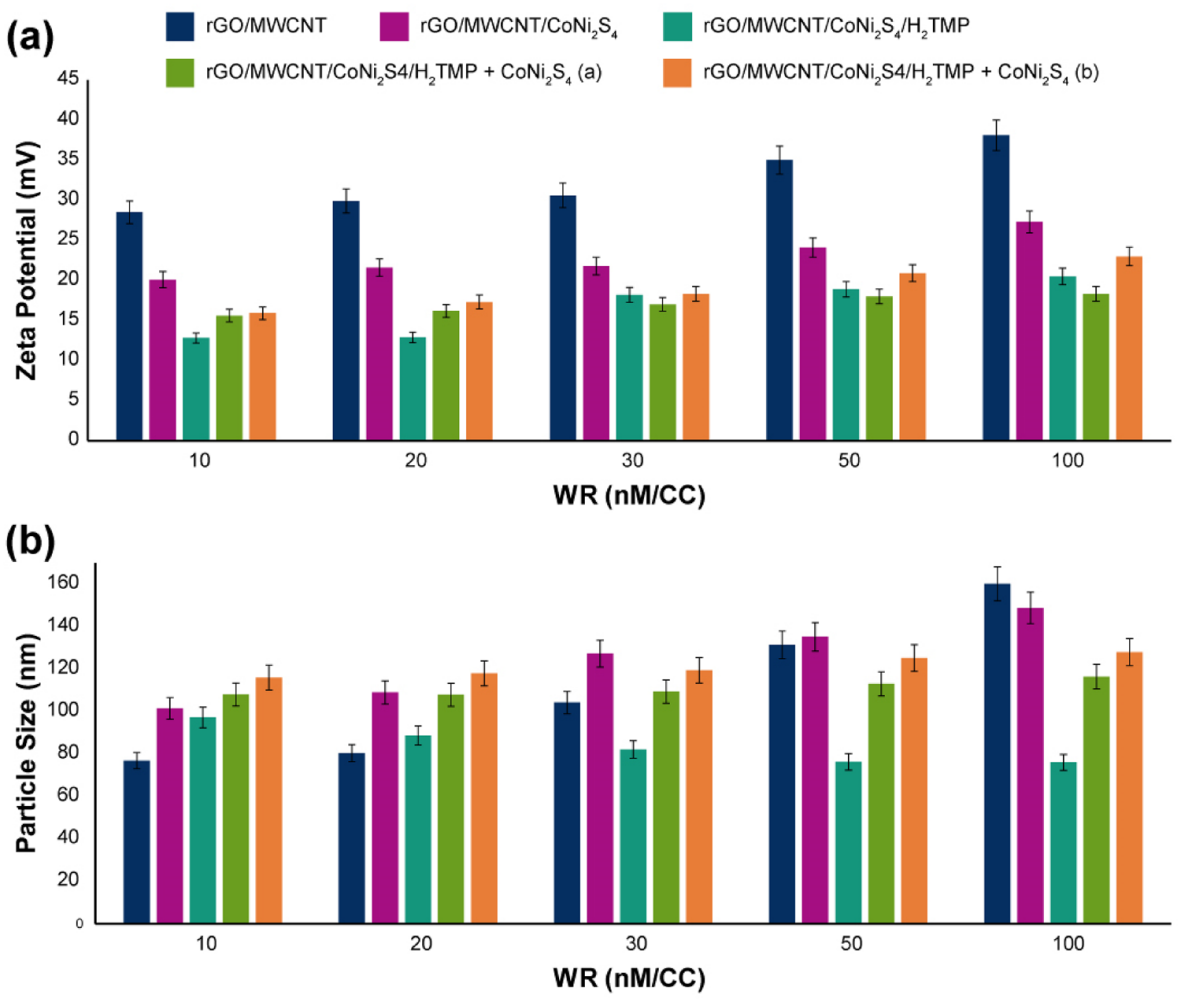
(**a**) The zeta potential results of the synthesized 3D nanocomposite along with each compartment of that and by addition of the excess amount of CoNi_2_S_4_ (through blending with the nanocomposite) with (**a**) 10 mg mL^−1^ and (**b**) 20 mg mL^−1^ concentrations; and (**b**) the particle size results of the synthesized 3D nanocomposite along with each compartment blended with CRISPR/Cas9 of that and by addition of the excess amount of CoNi_2_S_4_ (through blending with the nanocomposite) with (**a**) 10 mg mL^−1^ and (**b**) 20 mg Ml^–1^ concentrations. One point regarding the zeta potential and particle size is that they were investigated in the presence of CRISPR/Cas9 and its biological matrix.

To optimize the synthesized 3D nanocomposite for the gene delivery application, the role of size and zeta potential should be investigated. In this regard, the zeta potential of the 3D nanocomposite was evaluated (Fig. 6a), and the zeta potential of the 3D synthesized nanocomposite was recorded about 20 mV in the top. All of the data indicates that by increasing the weight ratio (WR) of nanomaterial (nM) to CRISPR/Cas9 (CC), the zeta potential naturally shifted to more positive numbers, and this shift is in the less slope in the presence of H_2_TMP and CoNi_2_S_4_ due to their chemical nature. Furthermore, the role of active species nanoparticles, CoNi_2_S_4_, in trends of zeta potential was investigated and revealed that by addition of 10 and 20 mg mL^−1^ of this part to the 3D nanocomposite, zeta potential increased a little in the range of 10–20 weight ratio of nanomaterial to CRISPR/Cas9, however, above of this range (30–100 WR of nM/CC), a slight decrease in the zeta potential was observed which would be because of the presence of H_2_TMP on the surface that has been covered the surface of the carbon-based nanomaterial completely. The CoNi_2_S_4_ helps that to decrease the zeta potential. Based on the literature^86–89^, these results are in good agreement with the potentially active species in delivering CRISPR/Cas9. However, the size of this nanocomposite should analyze as well. To investigate and assess the potential of interaction between each compartment of the nanocomposite and different types of that, they were blended with the CRISPR/Cas9, and the particle size of them was evaluated (Fig. 6b). It should be noted that some parts of the nanocomposite, including the rGO/MWCNT does not feel capable of making strong interactions with the CRISPR/Cas9. However, they can be able to physically interact with genetic materials. For gene delivery applications, the final carrier’s size is significant due to the critical mechanisms that should be done^90–92^. For CRISPR/Cas9 delivery, the size of the final non-viral gene delivery vector with the CRISPR/Cas9 should be below 200 nm, and in some cases, below 120 nm would lead to significant results in terms of especially endosomal escape mechanism^93–95^. The results of the particle size of this study showed by increasing the WR of nM to CC from 10 to 100 for rGO/MWCNT and rGO/MWCNT/CoNi_2_S_4_, the size of the final nanoplex increased from ⁓ 80 to ⁓ 160 nm and from ⁓ 100 to ⁓ 140 nm, respectively. This is completely normal due to the rising physical interaction of the carbon-based nanomaterial with the CRISPR/Cas9 by increasing the WR that leads to increasing the particle size. However, the results of the rGO/MWCNT/CoNi_2_S_4_/H_2_TMP showed completely different evidence; the particle size decreased from ⁓ 100 nm to below ⁓ 80 nm, which is an observation for a different mechanism. In this regard, the hypothesis is that by increasing the WR of the nanocomposite, the aggregations decrease substantially, which is in agreement with microscopy results. As a result, the net WR of the nanocomposite and in the following, the particle size decreased substantially. This phenomenon would be because of large porphyrin groups on the surface of the nanocomposite, which could increase the J-aggregation between the porphyrins and decrease the total aggregations between the carbon-based nanomaterials.

**Figure 7:**
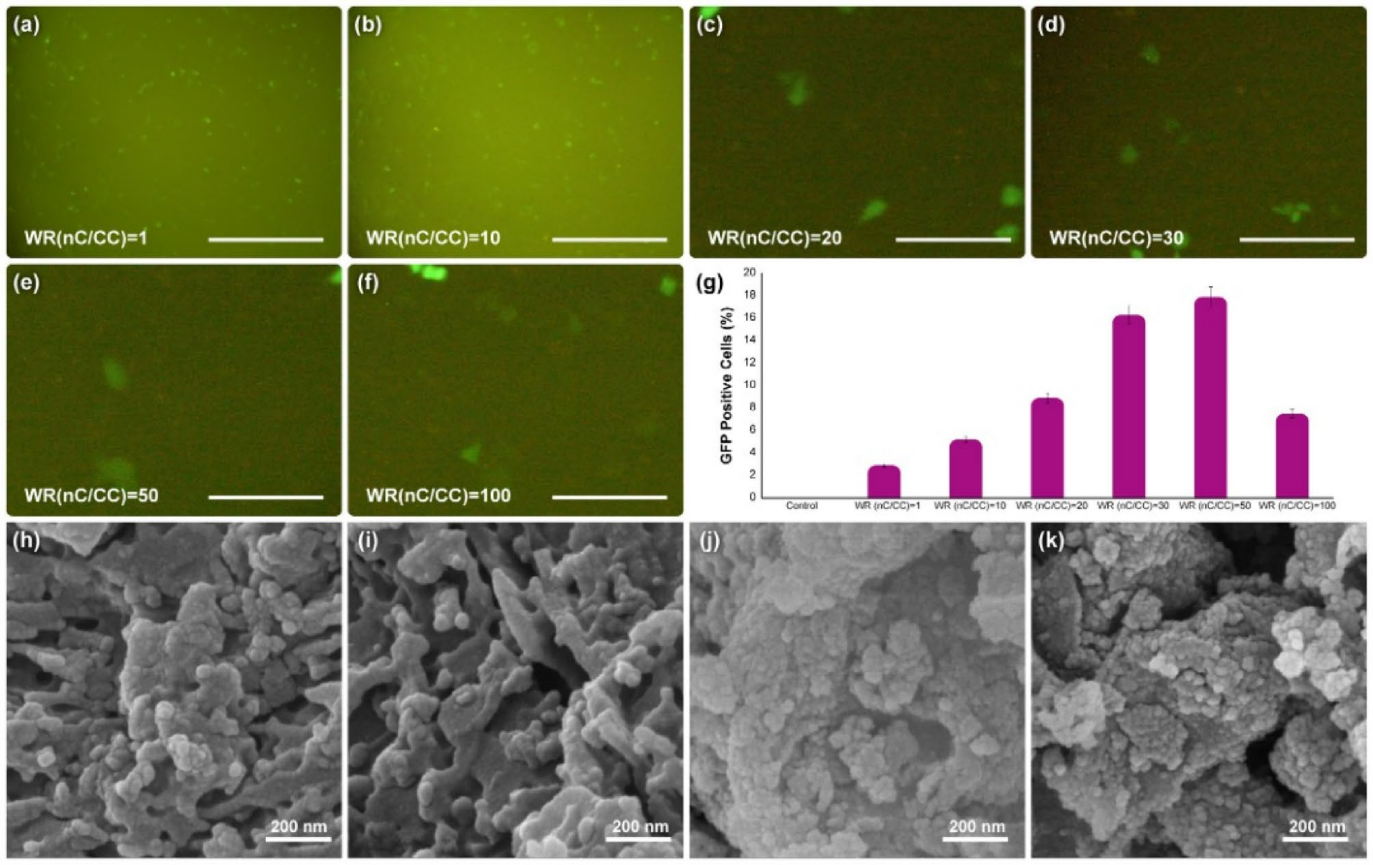
The results of (**a**–**f**) 2D fluorescence microscopy, and (**g**) GFP positive cells for the synthesized nanocomposite at different WR’s of nanocomposite (nC)/CRISPR/Cas9 (CC) on HEK-293 cell line. The data indicate the 2D fluorescence microscopy and EGFP read are presented as the mean (± SD) from three independent experiments. The Scale bar of (**a**,**b**) is 50 µm, and for (**c**–**f**) is 10 µm. The normal (**h**–**i**) FESEM analysis of the synthesized 3D nanocomposite degraded in the presence of different concentrations of hydrogen peroxide (same as the procedure conducted and shown in Fig. 3), and the normal (**j**–**k**) FESEM analysis of the synthesized 3D nanocomposite were removed from the cell culture after the pCRISPR gene transfection experiments.

In this study, pCRISPR expressing GFP was applied as a receptor to evaluate the synthesized nanocomposite’s capability in terms of in vitro gene delivery for the HEK-293 cell line. It should be noted that our goal was to design a new type of interaction in the gene delivery systems. Therefore, the only important objective for us is to provide and represent an acceptable GFP expression on the cell lines. Also, the aim of this study is the same as our previous study on the 2D layered double hydroxide study, with some advanced modifications^11^. Fluorescent microscopy methods were used to investigate the EGFP expression in HEK-293 cells at different weight ratios of the synthesized nanocomposite to pCRISPR to study gene transfection efficiency (Fig. 7a–g). Based on the results, by increasing the WR’s of nC to CC from 1 to 20, the transfection efficiency of GFP increased from 2.9 to 8.9%, which is a significant slope for this purpose. By increasing the WR’s of nC to CC from 20 to 30, another peak is conquered by increasing up to two-fold at 16.3%. However, by increasing this WR’s from 30 to 50, just about 1.6% added to the EGFP and reached to 17.9%. Interestingly, by doubling this ratio (50) to 100, a considerable decline in the GFP positive cells observed (declined to 7.5%), which would be because of increasing the aggregations of the nanocomposite and each part of that, especially the porphyrins together, that resulted in the destruction of cell walls. In this study, investigation of the genetic material release into the targeted cell was not important was not studied. So, more optimizations as well as critical investigations in order to adjust these types of interactions for possible gene delivery systems are mandatory. Furthermore, the morphology of the synthesized 3D nanocomposite was investigated via normal FESEM (Fig. 7h–k). The results showed that the nanostructure of the nanocomposite is wholley deformed, and as in the previous microscopic images, the thin and long carbon strands of the nanotubes were not observed. Still, here they can be seen very irregularly. Although multiple washes have been performed, a cell layer is observed on the surface that results from the destruction of cell walls. This study was designed based on the concept of chemical/physical interactions between the host (nanocomposite) and guest (pCRISPR). Therefore, these host–guest interactions lead to the acceptable EGFP, and could be considered as the next generation of any host–guest biomedical applications including drug delivery, biosensors and tissue engineering. Based on the literature^96–99^, there are several reports regarding the use of different nanomaterials and nan-viral nano-vectors in order to facilitate or generate a suitable interaction between the cargo (drug andor gene) and nanocarrier; however, this study revealed a new type of host–guest interaction between these materials, and showed very promising results. After the gene transfection procedure, the surface of the nanocomposite was studied with elemental mapping (Figure S4), and the results showed that the carbon-content is still low due to the coverage with the cobalt- and nickel-based nanomaterial, and the presence of cobalt, nickel and sulfur is still high. However, is some parts, the coverage with the inorganic components decayes, which is normal because of the cellular conditions, and the FESEM images confirmed the results as well. In addition, based on the literature (Table 2), till now, there is no report regarding the use of one-pot and easy synthesized carbon-based nanomaterial in the delivery of pCRISPR in assistance of EGFP. Therefore, this study could be considered as the cutting-edge result for the upcoming studies.

**Table 2:** A literature survey on the functionalized and modified carbon-based nanomaterials for gene delivery applications.

The category	Used materials	Notes	References
SWCNT	NH_2_-PEG-COOH, SWCNT-COOH, Aptamer, shRNA, Bcl-xL, PEI	Expensive method, not applicable for clinical trials, used for the transfection of Bcl-Xl shRNA	^100^
	PEG-NH_2_, SWCNT-COOH	Not applicable for clinical trials, used for the transfection of different range of genetic material, non-covalent modifications and attachements	^101^
	TERT siRNA, SWCNT, modified groups -CONH	Expensive carrier, not easy to produce and scale up, used to transfecr siRNA, used in the suppression of TERT expression approaches	^102^
	Succinate, SWCNT modified with PEI (different kDa’s)	Chemical bindings to the modifiers were selected, not green approach	^103^
	MAM2 siRNA, DSPE-PEG-Amine functionalized, SWCNT	Used to transfer siRNA-based genetic material, not green approach, not easy synthesis method	^104^
	hTERT siRNA, SWCNT, NGR peptide, PEI	Used to transfer siRNA-based genetic material, not green approach, not easy synthesis method	^105^
MWCNT	CdTe QDs, PAMAM, MWCNT, PEI, PDDA, Chitosan	Used electrostatic approach for the gene transfection, not green, not applicable for scale up for clinical trials	^106^
	Glycidyl trimethylammonium chloride siRNA, Dendron, MWCNT	Used electrostatic and chemical approach for the gene transfection, not green, not applicable for scale up for clinical trials	^107^
	siRNA, PAA, PEI, MWCNT	Used chemical and physical interaction for the immobalizations, not green, not applicable for clinical trials	^108^
	Cy3, MWCNT, pGL-3, PAA	Used a novel approach for the intracellular evaluation of the carrier using Cy3 labeled pGL-3	^109^
Graphene	PEI, pDNA, PLGA, oxidized graphene	Used electrostatic interaction for the gene delivery, Not green, Not applicable for clinical trials	^110^
	Graphene oxide, PEI, PSS, Adriamycin	Co- and simultaneouse delivery of both anti-miR-21 and Adriamycin, showed acceptable endosomal escape, not easy and green and cost-effective synthesis method	^111^
	Quantum dots, graphene, pDNA, MPG-2H1 chimeric peptide	Used graphene quantum dots functionalized with not cost-effective linkers, showed successful endosomal escape and cellular nuclear targeting	^112^
	LNA-m-MB, graphene, PEI	Both chemical and physical interactions, acceptable endosomal escape and transfection efficiency	^113^
	siRNA, EGFP, oxidized graphene	Great transfection efficiency, not easy to scale up, may have significant cytotoxicity in the clinical trials	^114^
	pCRISPR, EGFP, CoNi_2_S_4_, graphene, ZnO	One-pot and easy synthesis method, acceptable transfection efficiency, acceptable endosomal escape ability, superior stability, can be scale up for clinical trials	This work

Generally, this study was focused on the investigation of the new type of host–guest interaction for potential biomedical applications. In this manner, the host (nanocomposite) has several active sites including CoNi_2_S_4_, Graphene, MWCNT and porphyrin. Each one can interact with the guest (pCRISPR) in a chemical andor physical way. The CoNi_2_S_4_ compartment can be able to interact from the spatial approach, in order to capture the domain of the genetic material on its semi-cylindric shape. Also, graphene can be able to interact with the genetic material with its π–π interactions/aggregations along with the ability to provide the in situ growth of CoNi_2_S_4_. And at last, the porphyrin rings can be used as both stimuli-responsive agent for the both biosensor and gene delivery applications. This study proved that all of these factors affect each other, and enhance/improve the biosensor and gene delivery applications via their synergistic effects.

## Conclusion

Based on the aim of this study, a one-pot and cost-effective synthesis method for a carbon-based nanocomposite, rGO/MWCNT/CoNi_2_S_4_/H_2_TMP, was developed, optimized, and characterized. This nanocomposite showed interesting new and promising interaction with the genetic material. The surface morphology of the synthesized nanocomposite was studied carefully, and a chemicophysical relationship between the surface morphology of the nanocomposite, and each of the compartments of that with the biosensor and gene delivery applications was observed. In this case, an enhanced and extraordinary biosensor activity for in situ and real-time monitoring of hydrogen peroxide from living cells with the limit of detection of 20 µM was observed, which is a record by itself. Furthermore, for the first time, entirely physical interaction with the CRISPR/Cas9 was observed microscopically.The GFP transfections were recorded up to 17.9% by just physical interactions via their cracks and different step sizes, which would be considered as a revolution in gene delivery applications. More investigations should be conducted in order to optimize the results and applying them in clinical trials.

## Supplementary Information

Supplementary Information
