# Author Correction: Bio-multifunctional noncovalent porphyrin functionalized carbon-based nanocomposite

**DOI:** 10.1038/s41598-023-33771-2

**Published:** 2023-05-03

**Authors:** Navid Rabiee, Mojtaba Bagherzadeh, Amir Mohammad Ghadiri, Yousef Fatahi, Nafiseh Baheiraei, Moein Safarkhani, Abdullah Aldhaher, Rassoul Dinarvand

**Affiliations:** 1 Department of Chemistry, Sharif University of Technology, Tehran, Iran; 2 Department of Pharmaceutical Nanotechnology, Faculty of Pharmacy, Tehran University of Medical Sciences, Tehran, 14155-6451 Iran; 3 Nanotechnology Research Center, Faculty of Pharmacy, Tehran University of Medical Sciences, Tehran, 14155-6451 Iran; 4 Universal Scientific Education and Research Network (USERN), Tehran, 15875-4413 Iran; 5 Department of Anatomical Science, Faculty of Medical Sciences, Tarbiat Modares University, Tehran, Iran

Correction to: *Scientific Reports* 10.1038/s41598-021-86119-z, published online 23 March 2021

The original version of this Article contained errors in the Results section resulting from an incomplete revision of the text to reflect changes in the figures during review. Figure legends and the Results section were corrected accordingly.

As a result, in the Results section, under the subheading ‘In situ and real-time monitoring of hydrogen peroxide from the living cell’, the original legend of Figure 4

“Fluorescent image of the cells seeded on the nanocomposite after 16 h, for HEK-293 cell line (**a**,**b**) and PC12 cell line (**c**,**d**). (**b**,**d**) represents the fluorescence image after + 1 s after (**a**,**c**) captures. Fluorescence emission spectra of the synthesized 3D nanocomposite in the presence of different concentrations of PMA added to the HEK-293 cell line (**e**). Fluorescence emission spectra of the synthesized 3D nanocomposite in the presence of different concentrations of PMA added to the PC12 cell line (**f**). 2D (**g**) and 3D (**h**) AFM images and (k,i) FESEM images of the synthesized 3D nanocomposite after removing from the PC12 culture and exposure to the hydrogen peroxide; 2D (**i**) and 3D (**j**) AFM images and (**m**,**n**) FESEM images of the synthesized 3D nanocomposite after removing from the HEK-293 culture and exposure to the hydrogen peroxide.”

now reads:

“Fluorescence emission spectra of the synthesized 3D nanocomposite in the presence of different concentrations of PMA added to the HEK-293 (**a**) and PC12 (**b**) cell lines. 2D (**c**) and 3D (**d**) AFM images and (**g** and **h**) FESEM images of the synthesized 3D nanocomposite after removing from the PC12 culture and exposure to the hydrogen peroxide; 2D (**e**) and 3D (**f**) AFM images and (**i** and **j**) FESEM images of the synthesized 3D nanocomposite after removing from the HEK-293 culture and exposure to the hydrogen peroxide.”

Furthermore, the text about these data under the same subheading,

“Figure 4 shows the fluorescence image of the cells that have been seeded on the synthesized 3D nanocomposite, rGO/MWCNT/CoNi_2_S_4_/H_2_TMP, after 16 h (Fig. 4a,c) and 16 h + 1 s (Fig. 4b,d). It has been observed that most of the HEK-293 cells are alive, but the ratio of dead cells of the PC12 cell line is much higher than HEK-293.”

now reads:

“Figure 4 shows the fluorescence spectroscopy results of the nanocomposites that have been exposed to the cells and their hydrogen peroxides released. If the cells do not have significant and suitable adhesions, therefore, the fluorescence spectra do not show trends of the decline of emission in the presence of a different concentration of added PMA.”

Additionally, the in-text citations to the panels of Figure 4 under the same Results subheading have been updated to account for the change of panel numbers. Therefore,

“To monitor the hydrogen peroxide release from living cells, the phorbol 12-myristate 13-acetate (PMA) was used as a standard and routine stimulus agent to express hydrogen peroxide^73–75^. By adding this stimulus agent, PMA, a series of signaling pathways activated, and the hydrogen peroxide released from the living cells in a non-controlled manner. The hydrogen peroxide release mechanism is based on the oxidation of oxygen by NADPH oxidase and generating the O_2_^−^. To optimize the release of hydrogen peroxide upon the addition of PMA, a series of studies were designed and optimized based on the presence of HEK-293 and PC12 cell lines. Based on the results on the HEK-293 cell line (Fig. 4e), upon addition of 120 ng mL^−1^ of PMA to the cultured cell lines on the 3D nanocomposite, a distinctive decline in the fluorescence spectra of the media was observed, which was an equivalent phenomenon same as the addition of 20 µM of hydrogen peroxide. The maximum fluorescence intensity of the 3D nanocomposite decreased up to below a third compared to the absence of PMA. Furthermore, the PC12 cell line (Fig. 4f) represents not too much decrease in the presence of PMA, which could be considered a promising result based on the designing of a semi-selective fluorescence biosensor towards the HEK-293 cell line. Also, the maximum decrease of the fluorescence intensity in the presence of up to 260 ng mL^−1^ of PMA is equivalent to 40 µM of hydrogen peroxide. Therefore, this biosensor is not sensitive to both of the PC12 and HEK-293 cell lines. However, this is sensitive and accurate for analyzing released hydrogen peroxide from the HEK-293 cell line. Furthermore, the morphology of the synthesized 3D nanocomposite was investigated after removing from the PC12 and HEK-293 cultures via AFM (Fig. 4g–j) and FESEM (Fig. 4k–n), which have been shown that the surface morphology does not change significantly. However, the bulk breaks down into smaller pieces because of the in situ hydrogen peroxide the the nanocomposite’s cellular environment. Interestingly, by comparing the results of Fig. 4g–j with Fig. 3f–i, it is understood that in the presence of pure hydrogen peroxide, the surface has changed.”

now reads:

“To monitor the hydrogen peroxide release from living cells, the phorbol 12-myristate 13-acetate (PMA) was used as a standard and routine stimulus agent to express hydrogen peroxide^73,74,75^. By adding this stimulus agent, PMA, a series of signaling pathways activated, and the hydrogen peroxide released from the living cells in a non-controlled manner. The hydrogen peroxide release mechanism is based on the oxidation of oxygen by NADPH oxidase and generating the O_2_^−^. To optimize the release of hydrogen peroxide upon the addition of PMA, a series of studies were designed and optimized based on the presence of HEK-293 and PC12 cell lines. Based on the results on the HEK-293 cell line (Fig. 4a), upon addition of 120 ng mL^−1^ of PMA to the cultured cell lines on the 3D nanocomposite, a distinctive decline in the fluorescence spectra of the media was observed, which was an equivalent phenomenon same as the addition of 20 µM of hydrogen peroxide. The maximum fluorescence intensity of the 3D nanocomposite decreased up to below a third compared to the absence of PMA. Furthermore, the PC12 cell line (Fig. 4b) represents not too much decrease in the presence of PMA, which could be considered a promising result based on the designing of a semi-selective fluorescence biosensor towards the HEK-293 cell line. Also, the maximum decrease of the fluorescence intensity in the presence of up to 260 ng mL^−1^ of PMA is equivalent to 40 µM of hydrogen peroxide. Therefore, this biosensor is not sensitive to both of the PC12 and HEK-293 cell lines. However, this is sensitive and accurate for analyzing released hydrogen peroxide from the HEK-293 cell line. Furthermore, the morphology of the synthesized 3D nanocomposite was investigated after removing from the PC12 and HEK-293 cultures via AFM (Fig. 4c–f) and FESEM (Fig. 4g–j), which have been shown that the surface morphology does not change significantly. However, the bulk breaks down into smaller pieces because of the in situ hydrogen peroxide and the nanocomposite’s cellular environment. Interestingly, by comparing the results of Fig. 4c–f with Fig. 3f–i, it is understood that in the presence of pure hydrogen peroxide, the surface has changed.”

Lastly, in the Results section, under the subheading ‘CRIPSR/Cas9 investigations’, the original legend of Figure 7,

“The results of (**a**–**f**) 2D fluorescence microscopy, and (**g**) GFP positive cells for the synthesized nanocomposite at different WR’s of nanocomposite (nC)/CRISPR/Cas9 (CC) on HEK-293 cell line. The data indicate the 2D fluorescence microscopy and EGFP read are presented as the mean (± SD) from three independent experiments. The Scale bar of (**a**,**b**) is 50 µm, and for (**c**–**f**) is 10 µm. The normal (**h**–**k**) FESEM analysis of the synthesized 3D nanocomposite removed from the cell culture after the pCRISPR gene transfection experiments.”

now reads:

“The results of (**a**–**f**) 2D fluorescence microscopy, and (**g**) GFP positive cells for the synthesized nanocomposite at different WR’s of nanocomposite (nC)/CRISPR/Cas9 (CC) on HEK-293 cell line. The data indicate the 2D fluorescence microscopy and EGFP read are presented as the mean (± SD) from three independent experiments. The Scale bar of (**a**,**b**) is 50 µm, and for (**c**–**f**) is 10 µm. The normal (**h**–**i**) FESEM analysis of the synthesized 3D nanocomposite degraded in the presence of different concentrations of hydrogen peroxide (same as the procedure conducted and shown in Figure 3), and the normal (**j**–**k**) FESEM analysis of the synthesized 3D nanocomposite were removed from the cell culture after the pCRISPR gene transfection experiments.”

The original Article has been corrected.

