# Supplementary material for: Bio-multifunctional noncovalent porphyrin functionalized carbon-based nanocomposite
Source: Sci Rep. 2021 Mar 23;11:6604. doi: 10.1038/s41598-021-86119-z (PMC7988124; doi:10.1038/s41598-021-86119-z)
Supplement: Supplementary file 1 — Supplementary Information [file 41598_2021_86119_MOESM1_ESM.docx]

**Supplementary Information**

**Bio-Multifunctional noncovalent porphyrin functionalized carbon-based nanocomposite**

Navid Rabiee^1^, Mojtaba Bagherzadeh^1,*^, Amir Mohammad Ghadiri^1^, Yousef Fatahi^2,3,4^, Nafiseh Baheiraei^5^, Moein Safarkhani^1^, Abdullah Aldhaher^1^, Rassoul Dinarvand^2,3^

1. Department of Chemistry, Sharif University of Technology, Tehran, Iran
2. Department of Pharmaceutical Nanotechnology, Faculty of Pharmacy, Tehran University of Medical Sciences, Tehran 14155-6451, Iran
3. Nanotechnology Research Center, Faculty of Pharmacy, Tehran University of Medical Sciences, Tehran 14155-6451, Iran
4. Universal Scientific Education and Research Network (USERN), Tehran 15875-4413, Iran
5. Tissue Engineering and Applied Cell Sciences Division, Department of Hematology, Faculty of Medical Sciences, Tarbiat Modares University, Tehran, Iran

*Corresponding author: Prof. Mojtaba Bagherzadeh; [bagherzadeh@sharif.edu](mailto:bagherzadeh@sharif.edu); +98(21)66165301


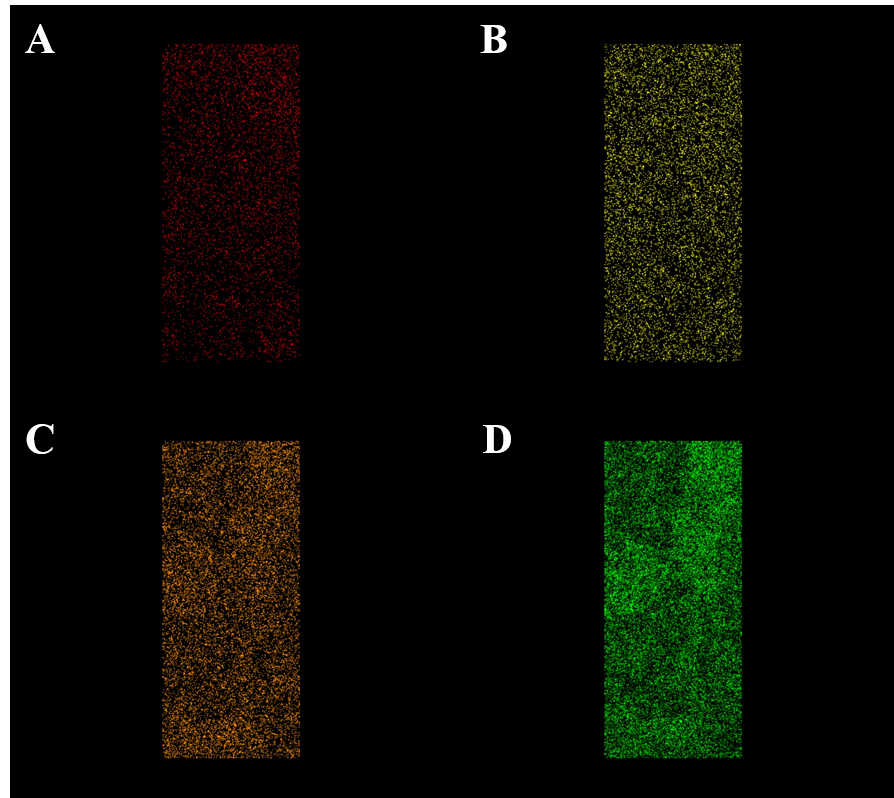


**Figure S1.** Elemental mapping for Carbon (A), Cobalt (B), Nickel (C) and Sulfur (D).


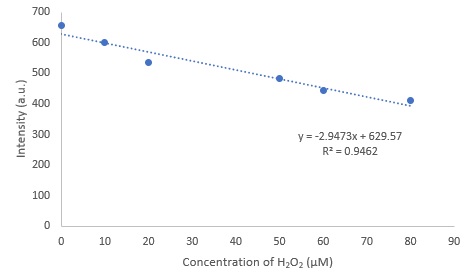


**Figure S2**. The relationship between the intensity decay and addition of more concentration of H_2_O_2_


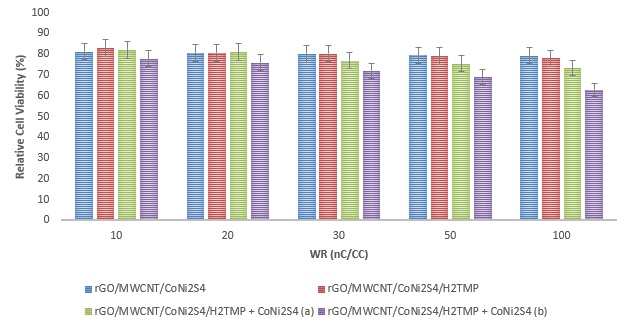


**Figure S3.** The results of the nanocomposites compartments-induced cytotoxicity assessed by LDH leakage assay via an underestimation process


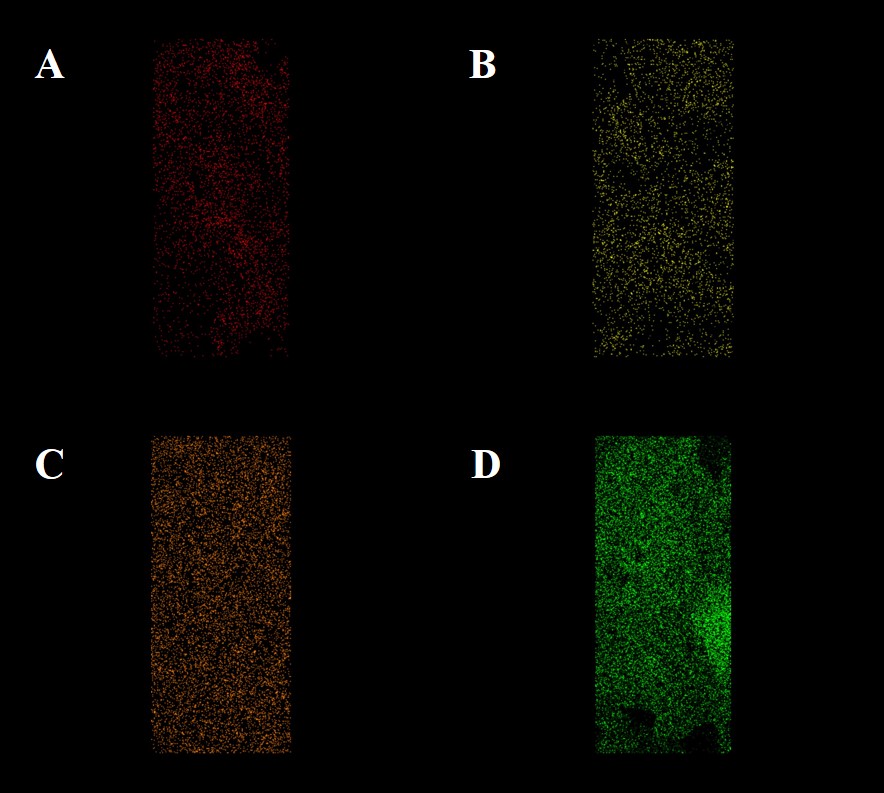


**Figure S4.** Elemental mapping for Carbon (A), Cobalt (B), Nickel (C) and Sulfur (D) after the gene transfection procedure.
